# Fluorescence radial fluctuation enables two-photon super-resolution microscopy

**DOI:** 10.3389/fncel.2023.1243633

**Published:** 2023-10-10

**Authors:** Motosuke Tsutsumi, Taiga Takahashi, Kentaro Kobayashi, Tomomi Nemoto

**Affiliations:** ^1^Biophotonics Research Group, Exploratory Research Center on Life and Living Systems, National Institutes of Natural Sciences, Okazaki, Japan; ^2^Research Division of Biophotonics, National Institute for Physiological Sciences, National Institutes of Natural Sciences, Okazaki, Japan; ^3^Nikon Imaging Center, Research Institute for Electronic Science, Hokkaido University, Sapporo, Japan

**Keywords:** two-photon microscopy, super-resolution, SRRF, *in vivo* imaging, spine morphology

## Abstract

Despite recent improvements in microscopy, it is still difficult to apply super-resolution microscopy for deep imaging due to the deterioration of light convergence properties in thick specimens. As a strategy to avoid such optical limitations for deep super-resolution imaging, we focused on super-resolution radial fluctuation (SRRF), a super-resolution technique based on image analysis. In this study, we applied SRRF to two-photon microscopy (2P-SRRF) and characterized its spatial resolution, suitability for deep observation, and morphological reproducibility in real brain tissue. By the comparison with structured illumination microscopy (SIM), it was confirmed that 2P-SRRF exhibited two-point resolution and morphological reproducibility comparable to that of SIM. The improvement in spatial resolution was also demonstrated at depths of more than several hundred micrometers in a brain-mimetic environment. After optimizing SRRF processing parameters, we successfully demonstrated *in vivo* high-resolution imaging of the fifth layer of the cerebral cortex using 2P-SRRF. This is the first report on the application of SRRF to *in vivo* two-photon imaging. This method can be easily applied to existing two-photon microscopes and can expand the visualization range of super-resolution imaging studies.

## 1. Introduction

Morphological changes in synaptic adaptation during learning have given insights into essential mechanisms for short-term or long-term memory formation (Abraham et al., 2019). For example, long-term potentiation (LTP) of synaptic transmission in the hippocampus is critical for declarative memory (Corkin, 2022) and long-term depression (LTD) of synaptic transmission has been reported to contribute to cerebellar learning (Ito, 2001). In both LTP and LTD, the size, shape, and number of dendritic spines often dynamically change. The size of the dendritic spines is strongly related to the sensitivity of postsynaptic receptors for neurotransmitters (Matsuzaki et al., 2001). In addition, several morphological features of the dendritic spine neck, including its width and length, affect electrical signaling at synapses (Tønnesen et al., 2014). Thus, a proper visualization at the sub-micrometer level of neuron morphologies, including dendritic spines, is critical for understanding the mechanisms of memory formation and processes involved in higher brain functions. To assess the nanostructure of dendritic spines such as the width and length of the spine neck, spatial resolution of around 100 nm is required. Such detailed observation of morphological changes in neurons have been accomplished using electron microscopy (EM) of fixed tissues (Arellano et al., 2007), even though EM cannot be easily combined with functional assays. Recently, super-resolution imaging techniques have allowed visualization and quantification of the morphological changes of dendritic spines combined with functional assays in cultured neurons and tissues (Tønnesen et al., 2014; Kashiwagi et al., 2019). In addition, there are various reports on super-resolution imaging of finer morphologies in living brains (Pfeiffer et al., 2018; Willig, 2022).

Recently, there have been reports of successful super-resolution imaging in brain tissue using stimulated emission depletion microscopy (STED) or structured illumination microscopy (SIM) at 50–100 μm depth from the sample surface by adjusting the refractive index and scattering of the fixed sample to suppress spherical aberrations (Ke et al., 2016; Sawada et al., 2018). Further, combining two-photon excitation, STED, and adaptive optics enable observations at relatively deep regions in acute brain slices and *in vivo* brains, but still only down to 100 μm from the sample surface (Bethge et al., 2013; Pfeiffer et al., 2018; Bancelin et al., 2021). So far, the super-resolution microscopy techniques available have not allowed imaging in deeper layers due to optical limitations (Schermelleh et al., 2019). The increasing optical aberrations occurring while observing thick tissues or *in vivo* imaging degrade the excitation laser light beam, which expands the focal volume and worsens the spatial resolution and fluorescent signal. For STED or SIM, serious deterioration of the vortex or stripe pattern of the laser beam prevents the improvement of spatial resolution. Thus, visualizing neuronal morphology at the sub-micrometer level in the deep brain *in vivo* requires other technical approaches.

Here, we focused on super-resolution radial fluctuation (SRRF), a super-resolution technique that uses spatiotemporal fluorescence fluctuation analysis (Gustafsson et al., 2016). This method does not require parameters related to optical properties for processing. There is no need for dedicated optics for excitation or depletion of fluorescent probes. Furthermore, it does not require specific fluorescent dyes/proteins, since the SRRF can isolate fluorophores with higher density than existing single-molecule localization microscopy (SMLM), which makes it potentially compatible with various existing fluorescence microscopes. Thus, in this study, we used SRRF in combination with two-photon microscopy (2P-SRRF) to achieve a spatial resolution of around 100 nm *in vivo* observation. 2P-SRRF was evaluated its effects on spatial resolution, the possibility of imaging at deeper areas, and morphological reproducibility. In addition, we optimized the image processing parameters for suppressing artifacts. In conclusion, we successfully demonstrated high-resolution *in vivo* imaging of the deep cortex, something so far unamenable with existing super-resolution microscopy techniques.

## 2. Materials and methods

### 2.1. Preparation of *in vitro* samples

A GATTA-SIM nanoruler prepared slide (#SIM120B), an evaluation tool for the spatial resolution of super-resolution microscopy was purchased from GATTA quant (Gräfelfing, Germany). The sample consisted of 120 ± 5 nm rods made of DNA origami with Alexa Fluor 488 labeled ends and affixed to the surface of the coverslip.

For the preparation of the brain mimetic gel sample, Intralipid (Intralipid Infusion Solution 20%, Fresenius Carbi-Japan, Tokyo, Japan) at a final concentration of 1% (w/v) was mixed to prepare 2% (w/v) agarose gels to achieve a scattering coefficient (10 cm^−1^) similar to that of mouse biological brain cortex as reported previously (Urban et al., 2018). Further, 16% (w/v) sucrose water was also added to the gel solution to achieve a refractive index (RI = 1.36) (Ue et al., 2018; Yamaguchi et al., 2021) of mouse brain cortex. Before gel solidification, *φ*100 nm-YellowGreen beads (FluoSpheres carboxylate modified microspheres, 0.1 μm, yellow-green fluorescent, Invitrogen, MA, United States) were diluted 100-fold and embedded in the gel solution.

### 2.2. Animals

Adult transgenic mice expressing the fluorescent protein EYFP in excitatory neurons (Thy1-EYFP-H, hereafter referred to as H-line mice) (Feng et al., 2000) were bred and used for the experiments. Mice were housed at 22°C–24°C with a standard 12 h light–dark cycle and *ad libitum* access to water and a standard chow. All animal studies were carried out in accordance with ARRIVE guidelines and all animal care and experimental procedures were approved by the Institutional Animal Care and Use Committee of the National Institute of Natural Sciences and were performed according to the guidelines of the National Institute for Physiological Sciences (Approval No. 20A017 and 20A122).

### 2.3. Microscopes

Imaging was performed with an upright multiphoton microscope system Nikon A1R-MP^+^ (Nikon, Tokyo, Japan) equipped with a Ti: Sapphire laser source (MaiTai DeepSee, Spectra-Physics, Santa-Clara, CA) emitting near-infrared ultrashort laser light pulses and highly sensitive GaAsP-NDD detectors. A 25× long working distance water dipping objective (Apo LWD 25×/1.10 W, Nikon) and a 60× high numerical aperture (NA) water immersion objective (SR Plan Apo IR 60×/1.27 WI, Nikon) were utilized for imaging. The excitation wavelength was 950 nm, and fluorescence was acquired in the 500–550 nm range using a dichroic mirror and a fluorescence filter. The image pixel size was set to 104 nm/pixel in all cases except for the pixel size verification by adjusting of zoom factor and scanning image size. Further details on imaging conditions including scan speed are shown in Supplementary Table S1.

A SIM, Nikon N-SIM (Nikon) microscope with a 100× oil immersion objective (Apo TIRF 100×/1.49 Oil, Nikon) and sCMOS camera (ORCA Flash 4.0, Hamamatsu Photonics, Hamamatsu, Japan) was used for the comparison of 2P-SRRF in nanoruler and fixed brain slice images. The excitation wavelength was set to 488 nm. The pixel size was set to 32.5 nm/pixel. For fixed brain slice observation, 3D-SIM mode was used. Raw images for SIM reconstruction were acquired every 120° rotation. The grating pitch of structured illumination corresponded to the diffraction limit of light. Image stacks of optical sections were acquired with 200 nm Z-steps. 3D reconstructions of SIM images were performed using the processing algorithm V2.10 of NIS-Elements AR (Nikon).

### 2.4. Fixed brain slices

H-line mice were perfusion-fixed in 4% (w/v) paraformaldehyde in phosphate-buffered saline (PBS) under deep anesthesia by isoflurane inhalation, and whole brains were dissected. After additional fixation, coronal sections including the cerebral cortex were prepared using a vibratome (700smz, Campden Instruments, Leicestershire, United Kingdom) as 100 or 200 μm-thick. For the deep imaging demonstration and the pixel size verification, fixed brain sections of 100 μm-thick were mounted in PBS on a 35 mm glass bottom dish (*φ*27 mm No. 1S, Iwaki, Shizuoka, Japan) for observation. For spatial resolution comparison with SIM, 200 μm-thick fixed brain sections were treated with ScaleA2 solution [4 M urea, 10% (w/v) glycerol, and 0.1% (w/v) Triton X-100] at 37°C for 2 days for optical clearing of sample (Hama et al., 2011), mounted in ScaleA2 on a glass bottom dish, and the Z-stack images of same field of view was observed with two-photon microscopy and 3D-SIM. The comparisons of spatial resolution and reproducibility of images were performed on a single plane of the Z-stack images.

### 2.5. *In vivo* observation

A cranial window was opened by surgery using the open skull method in H-line mice (Holtmaat et al., 2009) under deep anesthesia by isoflurane inhalation. To suppress heartbeat-related tissue movement as far as possible, the cranial window was sealed with a double coverslip (No. 1S, Matsunami Glass, Kishiwada, Japan). To reduce optical aberrations, the mouse was held on a homemade three-axis adjustment stage (Kawakami et al., 2013) and observed while maintaining the stage angle to ensure the cover glass and the objective lens were parallel. After image acquisition, to register image misalignments caused by the animal heartbeat, registration was performed using the Fiji/ImageJ’s TurboReg plug-in (ver. 2.00).

### 2.6. SRRF processing

The NanoJ-SRRF plug-in (ver. 1.14 Stable1) of Fiji/ImageJ (ver. 1.53o) was used for SRRF processing. Processing was performed using 30 consecutively acquired images. The parameters of spatial analysis were ring radius = 0.1–2.0 (optimum value was chosen for each experiment), radiality magnification = 5, and axes in ring = 8. The temporal radiality average was used for temporal analysis. In the case of Z-stack images, SRRF processing was applied to each successive XY-image acquired at each depth followed by reconstruction of the Z-stack image. As the intensity values of the reconstructed images obtained by SRRF processing were real numbers, it was difficult to compare the intensity with other images without modification. Thus, normalized values were used to indicate the intensity of both images and intensity profiles.

### 2.7. Analyses

To evaluate the spatial resolution of 2P-SRRF, we used three different resolution criteria; precision of center-of-gravity determination, two-point resolution, and Fourier ring correlation (FRC) spatial frequency analyses. To analyze the precision of center-of-gravity, the full width at half maximums (FWHMs) of the fluorescence peaks were determined by curve fitting applying a Gaussian function on the intensity profile using Fiji/ImageJ. For the confirmation of the two-point resolution of 2P-SRRF, distances between the fluorescence peaks of nanorulers were measured and compared with those measured in the SIM images as the mean ± standard error. The FRC analyses were performed as previously reported (Nieuwenhuizen et al., 2013) using the BIOP plug-in of Fiji/ImageJ. The spatial frequencies at which the FRC curves reach the correlation value of 1/7 were used as the spatial resolutions.

To perform a parameter sweep of the ring radius for SRRF processing, the NanoJ-SQUIRREL plug-in of Fiji/ImageJ was utilized referring to the previous report (Culley et al., 2018).

## 3. Results

### 3.1. Confirmation of super-resolution 2P-SRRF imaging

**Figure 1:**
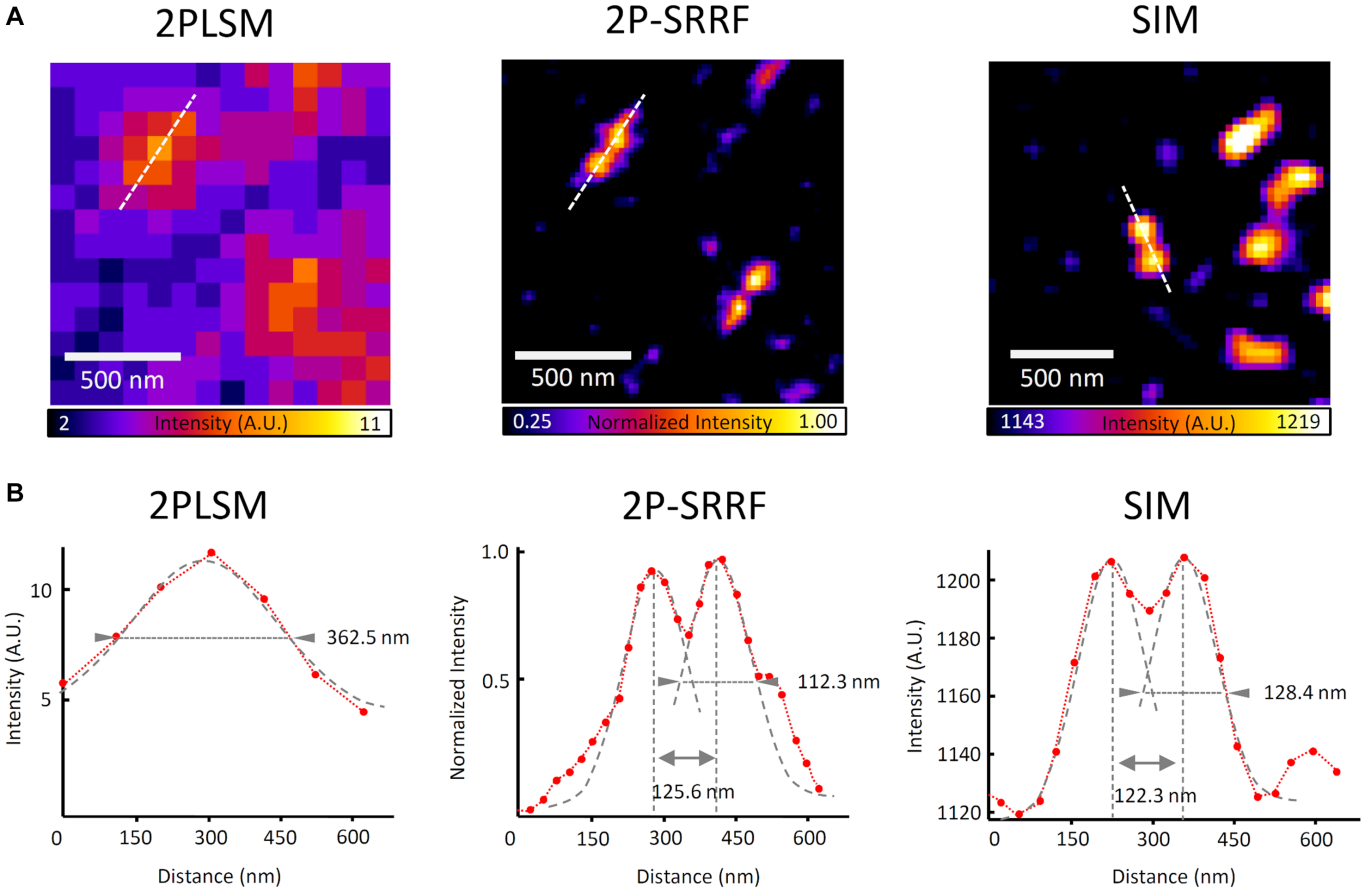
Confirmation of super-resolution image acquisition by 2P-SRRF. Comparison of two-photon super-resolution radial fluctuation (2P-SRRF) and structured illumination microscopy (SIM) images and the respective intensity profiles obtained from 120 nm nanorulers. Two-photon laser scanning microscopy (2PLSM) image shows the average of 30 consecutive images acquired by two-photon microscopy, and 2P-SRRF is the result of SRRF processing from the same images **(A)**. Intensity profiles of the nanoruler in **A**. **(B)** Each profile (red) indicates the fluorescence intensity distribution along the white dashed line indicated in the images. Arrowheads in the intensity profiles indicate the full width at half maximum (FWHM) determined by Gaussian curve fitting. The peak-to-peak distance was calculated as the distance between the vertices of two peaks determined by curve fitting.

To evaluate the effect on the spatial resolution of applying SRRF to two-photon microscopy, we used 120 nm-length nanorulers, which are mainly used for evaluating the two-point resolution of SIM. The samples consisted of 120 ± 5 nm rods made of DNA origami with fluorescently labeled ends. The bright spots of the nanoruler, visible as a single peak of width according to the diffraction limit with conventional two-photon microscopy (~360 nm), were separated into two peaks approximately 120 nm apart after applying 2P-SRRF (Figure 1; Supplementary Figure S1). The spatial resolution was similar to the SIM observations. Whether observed with 2P-SRRF (120.8 ± 1.4 nm) or SIM (121.5 ± 1.9 nm), the measured distances between the two peaks fell within the manufacturing error of the samples (*N* = 5, Supplementary Figure S1). These results indicate that applying SRRF to two-photon microscopy clearly improves spatial resolution and suggests the possibility of obtaining a spatial resolution as high as that obtained with SIM.

### 3.2. Verification of applicability to deep imaging

**Figure 2:**
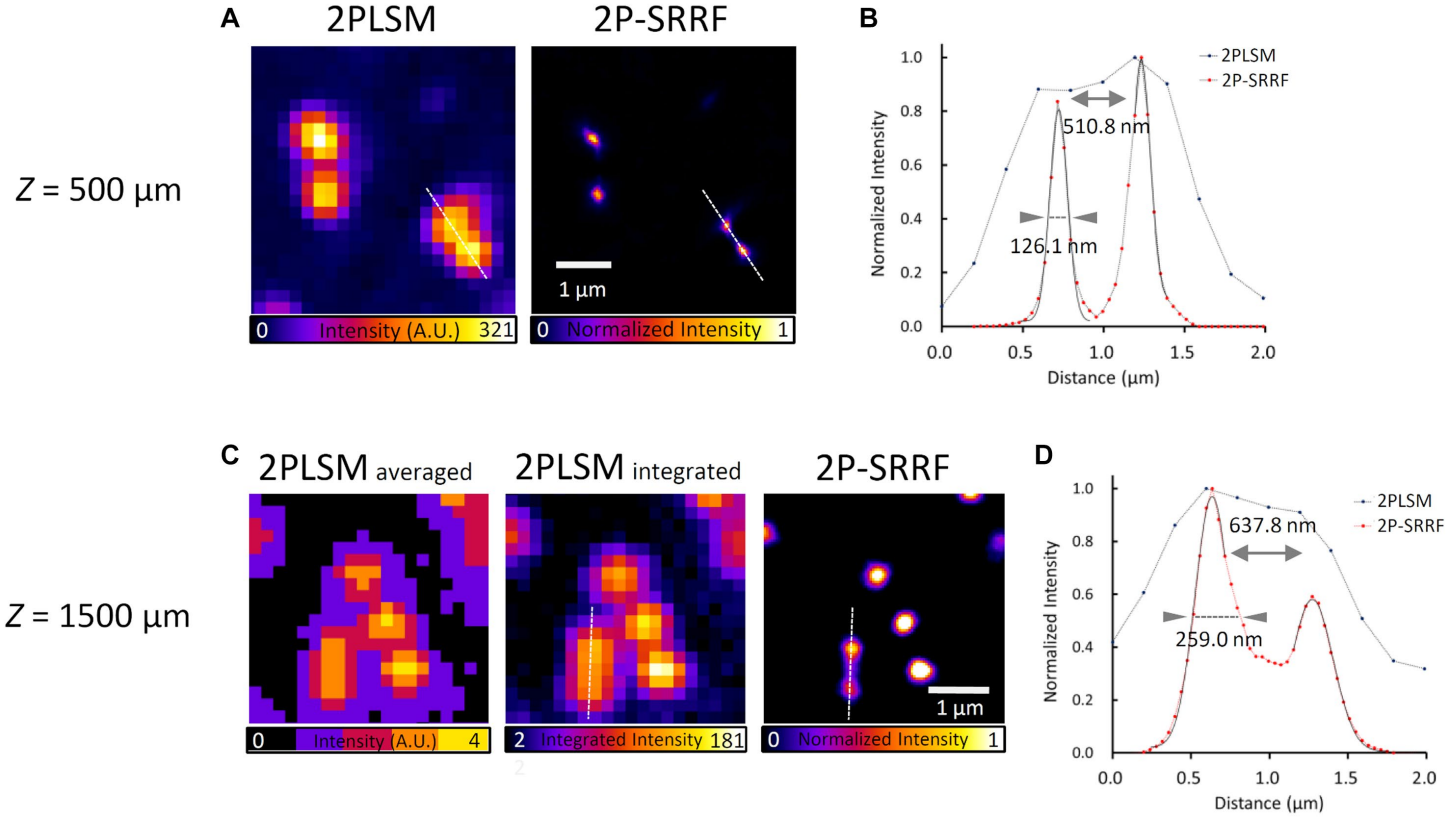
Verification of applicability to deep imaging. Fluorescent bead image and intensity profile at 500 μm depth in a mouse brain mimetic gel **(A,B)**. 2PLSM shows the average of 30 consecutive images acquired by two-photon microscopy; 2P-SRRF shows the result of SRRF processing from the same consecutive images. Fluorescent bead images and intensity profiles at 1500 μm depth in mouse brain mimetic gel **(C,D)**. **(B,D)** Intensity profiles of 2PLSM (blue) and 2P-SRRF (red) are plotted with intensities along the white dashed line in **A,C**. Arrowheads in the intensity profiles indicate the FWHM calculated by Gaussian curve fitting. The peak-to-peak distance was calculated as the distance between the vertices of two peaks.

Next, to verify the applicability of 2P-SRRF to deep imaging in tissues, we observed fluorescent beads embedded in a gel that mimics the refractive index and scattering coefficient of a living mouse brain (Figure 2). At a depth of 500 μm, 2P-SRRF could separate two peaks of fluorescent beads that could not be well-separated in the original two-photon image (Figure 2A). At 1,500 μm depth, very faint fluorescence could be obtained even using the full power of the excitation laser; however, with 2P-SRRF there was a clear improvement in spatial resolution (Figure 2B). On the other hand, FWHM of each peak in the 2P-SRRF image acquired at 1500 μm depth was broader than that at 500 μm depth. This was probably due to a lower signal-to-noise ratio (SNR), which reduced the precision of center-of-gravity determination in SRRF processing. Nevertheless, the applicability of 2P-SRRF to deep areas with strong scattering and reflection was confirmed. 2P-SRRF can be widely applicable to deep imaging in the range generally imaged by two-photon microscopy.

### 3.3. 2P-SRRF imaging in fixed brain slices

**Figure 3:**
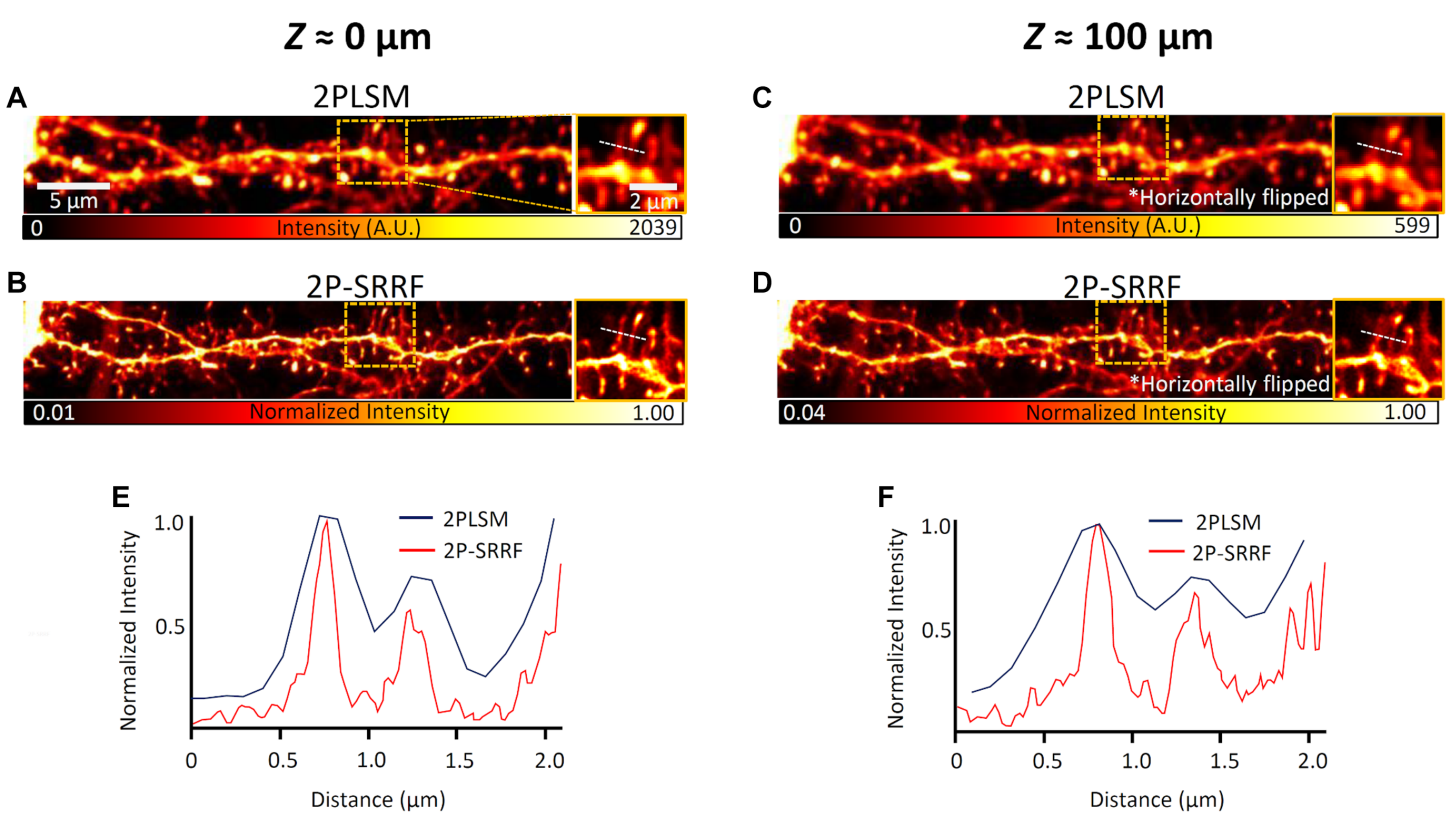
2P-SRRF in fixed brain slices. Comparison of images from the basal dendrites of layer 5 pyramidal cells from the same region near the surface **(A,B)** and at 100 μm depth **(C,D)** of coronal brain slices of Thy1-EYFP H mice. The 100 μm depth images were acquired by turning the slice over and looking through the tissue after acquiring the superficial image. The 2PLSM image is the average of those acquired consecutively by two-photon microscopy and the 2P-SRRF image was processed from the same consecutively acquired images. Each image also shows the maximum intensity projection of three planes of images acquired every 1 μm. Enlarged images of the region surrounded by orange dashed lines of images were shown on the right side of each image. Intensity profiles are shown as intensity distributions along the white dashed lines in the images of 2PLSM (blue) and 2P-SRRF (red) **(E,F)**.

Then, 2P-SRRF was tested on real brain tissue. The same field of view at layer 5 of the visual cortex of an H-line mouse was observed near the surface of a 100 μm-thick coronal brain slice and from the opposite end (equivalent to observation at 100 μm depth) (Figure 3). The microstructures of neuronal dendrites near the surface of the slice could be visualized when applying 2P-SRRF (Figure 3B). At 100 μm depth, the fine structures of dendritic spines were also visualized, and the spine necks could be distinguished well, even though no particular optical clearing of tissue was applied (Figure 3D). On the other hand, when the same sample was tested to be observed by SIM, even at depths of 50 μm, it was difficult to visualize tiny structures due to light scattering (Supplementary Figure S2).

**Figure 4:**
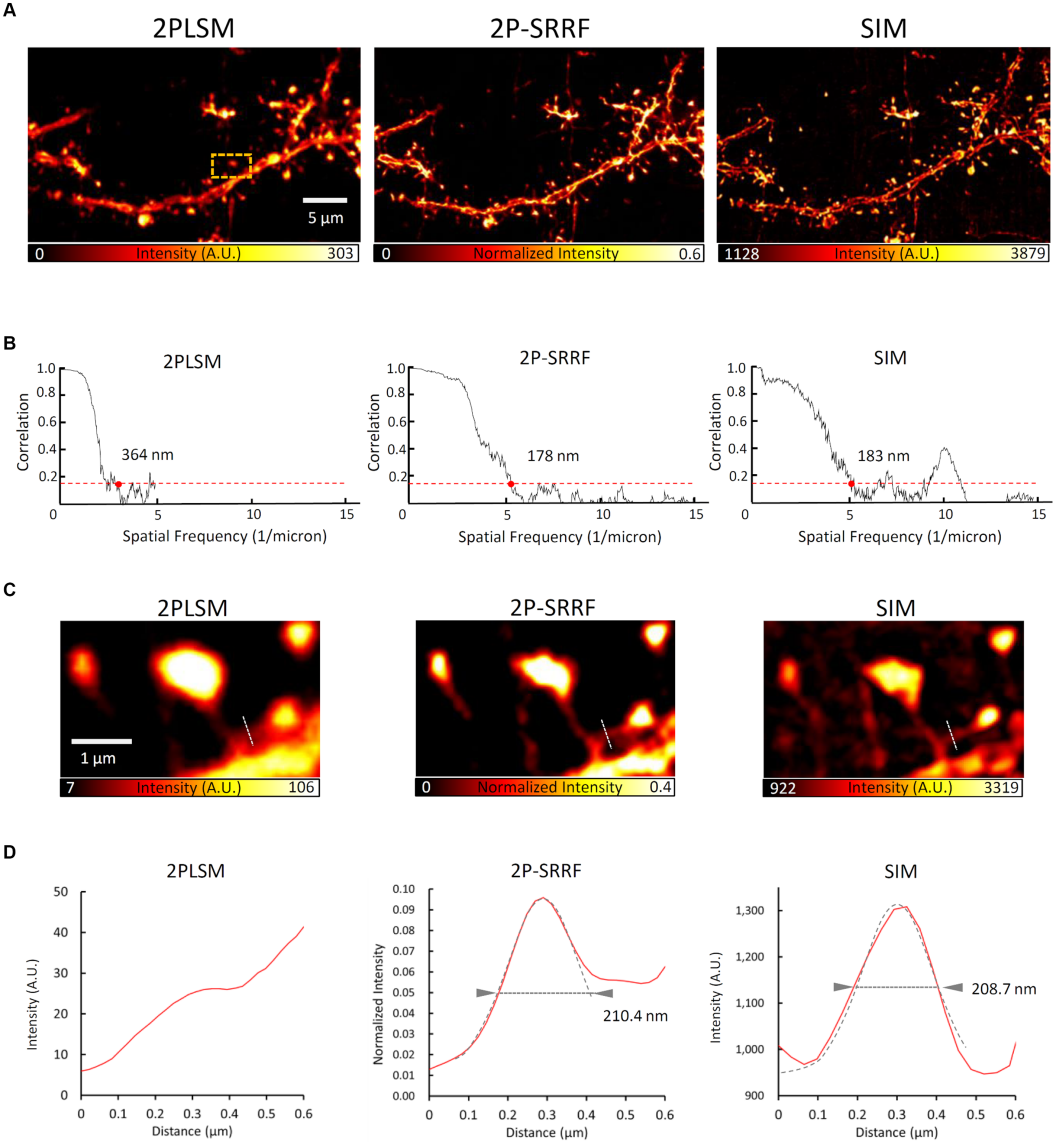
2P-SRRF vs. SIM in the same region of a fixed brain slice. Comparison of SIM and 2P-SRRF images in the same superficial region of Thy1-EYFP H mouse fixed coronal brain slices cleared with ScaleA2 solution. Averaged two-photon image (2PLSM), SRRF-processed (2P-SRRF), and maximum intensity projected (2.5 μm thick) Z-stack 3D-SIM images of same basal dendrites of layer 5 pyramidal cells in fixed brain cortex slices **(A)**. Plots of Fourier ring correlations for each image in **A**. **(B)** The 1/7 value, which reflects spatial resolution, is indicated by red dots. Enlarged image of the region surrounded by an orange dashed line in **A**. **(C)** Intensity profile of each image along the white dashed line in **C**. **(D)** Arrowheads in the intensity profiles indicate the FWHM calculated by Gaussian curve fitting.

In addition, to evaluate the morphological reproducibility and spatial resolution of 2P-SRRF, we compared 2P-SRRF and SIM images of another fixed brain slice (Figure 4). The same basal dendrites of cortical layer 5 pyramidal neurons located near the surface of the slice treated by ScaleA2 clearing were observed using both microscopy techniques. Comparing the images obtained by SIM with those by 2P-SRRF, the dendrite morphology was almost identical (Figure 4A). The analysis of frequency components in the image by FRC showed that the spatial resolution of the 2P-SRRF image (~178 nm) was approximately twice as high as that of the original two-photon image (~364 nm), and comparable to that of SIM (Figure 4B). Furthermore, comparing the morphology of a specific dendritic spine showed that the fine spine neck, buried in the fluorescence of the dendrite shaft in the original two-photon image, could be separated and visualized in 2P-SRRF as well as SIM (Figures 4C,D). These results indicate that 2P-SRRF can be applied to actual biological samples with morphological reproducibility and spatial resolution equivalent to SIM. Together with the above deep imaging results, 2P-SRRF might offer a spatial resolution comparable to existing super-resolution microscopes for *in vivo* deep imaging.

### 3.4. Improvement of morphological reproducibility and spatial resolution by optimizing processing parameters

We performed an exhaustive comparison and optimization of the imaging conditions of the original images and SRRF processing parameters in 2P-SRRF. As an example, we have assessed the effect of pixel size on the spatial resolution and morphological reproducibility of reconstructed super-resolution images. The evaluation was carried out using the same method as in Figure 4. Focusing on the same dendrite, we compared the width of the spine neck and FRC of 2P-SRRF images and original two-photon images acquired with various pixel sizes (Supplementary Figure S1). For a pixel size of 104 nm/pixel, the 2P-SRRF image showed the narrowest spine neck shape, with a width of ~173 nm. A pixel size of approximately 100 nm/pixel satisfies the Nyquist frequency (~180 nm) of this optical condition and is considered to contribute to the reproduction of finer morphological information in the SRRF processing. Nonetheless, smaller pixel sizes could not improve spatial resolution because of a decrease in the accuracy of determining the center of gravity of the fluorescence peaks due to the lower signal-to-noise ratio. Thus, when applying 2P-SRRF, it is important to set a pixel size that satisfies the Nyquist frequency while guaranteeing a high SNR.

We also compare the ring radius (*r*), one of the parameters for SRRF processing (Supplementary Figure S2). In this comparison, we evaluated the FRC distributions and reconstruction errors (which represent the fidelity of super-resolution images) of 2P-SRRF images by using NanoJ-SQUIRREL (Culley et al., 2018). For the neuron observation in fixed brain tissue same as Figure 4, *r* = 0.1 to 1.0 resulted in similar low errors of reconstruction, whereas larger ring radiuses increased the errors. In the FRC map comparison, high spatial frequency (low FRC) regions along the neural dendrites were observed when small *r* were applied. In this case, we chose *r* = 0.1 because it showed the lower minimum FRC value, the best continuity of a low FRC region, and relatively low reconstruction errors.

### 3.5. Application to *in vivo* imaging

**Figure 5:**
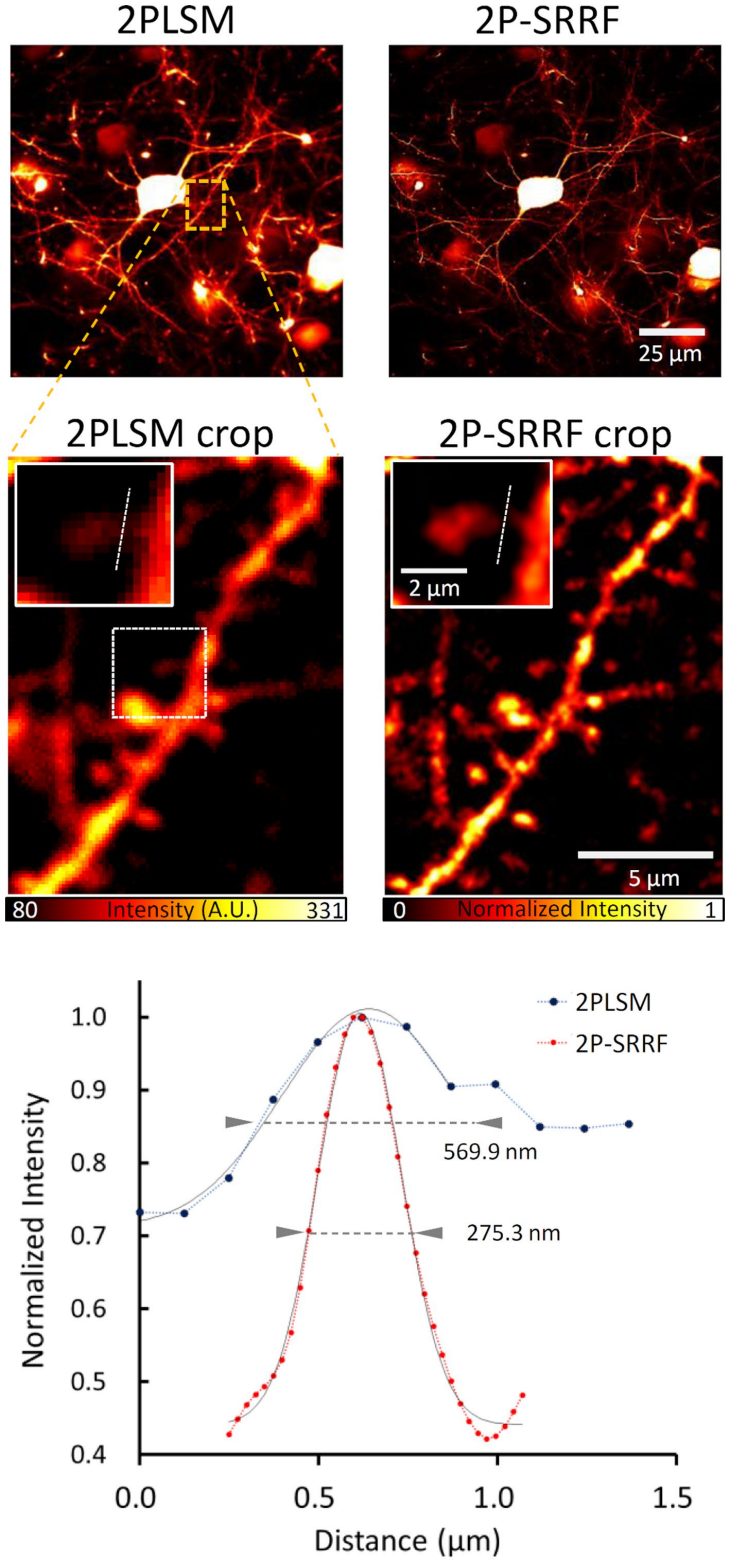
Application to *in vivo* imaging. Average *in vivo* two-photon (2PLSM) and 2P-SRRF images at high depth in the brain of Thy1-EYFP H mice. Crop images show an enlarged image of the area enclosed by the orange dashed line in the original figures. Further enlarged images of the single spine region surrounded by white dashed lines of crop images were shown in the insert. The lower panel shows the intensity profiles of 2PLSM (blue) and 2P-SRRF (red) along the white dashed lines in the insert images. Arrowheads in the intensity profiles indicate the FWHM calculated by Gaussian curve fitting.

Finally, we tested the application of 2P-SRRF to mouse *in vivo* brain imaging (Figure 5). Through a cranial window, pyramidal cells of layer 5 of the visual cortex were observed at 500 μm depth from the brain surface. After SRRF processing, finer dendrite structures could be observed. The width of a particular spine neck was approximately 275 nm, clearly improved from the spatial resolution of two-photon imaging (~570 nm).

Also, an *in vivo* timelapse observation was tested (Supplementary Figure S3). A dendrite was tracked for 5 min at a depth of 100 μm (2nd layer of the visual cortex) from the brain surface (Supplementary Figure S3A), and 2P-SRRF allowed us to stably observe fine spine neck structures (FWHM = 148 nm) that could not be recognized by conventional two-photon observation (Supplementary Figure S3B). The acquisition time for each single SRRF image was 2 s. These results suggest that 2P-SRRF could track the changes in spine morphology over several minutes with a time resolution of a few seconds.

During *in vivo* imaging, the mouse heartbeat caused blurring between consecutive image acquisitions, which interfered with the temporal correlation analysis in SRRF processing. To avoid this, in both *in vivo* observations, we applied image registration (automatic image alignment) to the source images. Without registration, visualization of the spine neck by 2P-SRRF was not possible (Supplementary Figure S3C).

## 4. Discussion

### 4.1. Improvements In spatial resolution of two-photon imaging utilizing image analysis-based techniques

In this study, we applied SRRF to two-photon deep imaging of brain tissue and uncovered nanostructures of dendritic spines with high-resolution, comparable to SIM (Figure 4). Further, we could observe detailed spine morphologies at deeper areas than previous reports (Bethge et al., 2013; Pfeiffer et al., 2018; Bancelin et al., 2021), at 500 μm brain depth *in vivo* (Figure 5). Previously, combining two-photon microscopy with deconvolution resulted in a high-resolution (approx. 230 nm) observation of thick fixed tissues (Kapsokalyvas et al., 2021). However, the target was a relatively thin area (<200 μm) of the transparent sample. Super-resolution optical fluctuation imaging (SOFI), based on a fluorescence correlation analysis similar to SRRF (Alva et al., 2022), was previously applied to two-photon light-sheet microscopy (Chen et al., 2016). Although SOFI successfully improved the spatial resolution, it required special flicking fluorescent dyes and a custom-made light-sheet microscope, making its *in vivo* application difficult. In addition, SRRF has been previously applied to two-photon microscopic imaging with nanodiamonds (Johnstone et al., 2019). However, this study focused mainly on the characterization of nanodiamonds as fluorescent probes and did not observe any biomaterials in actual living organisms or tissues. Thus, to our knowledge, the present study might be the first demonstration of two-photon super-resolution imaging in intact tissues and living specimens *in vivo* using SRRF.

Including SRRF and SOFI, fluorescence fluctuation-based super-resolution techniques have been developed and improved in recent years (Alva et al., 2022). Throughput, precision of center-of-gravity determination, and reliability of image reconstruction of these techniques have improved over the years. SR method based on the auto-correlation with two-step deconvolution (SACD) is also a newly reported super-resolution technique using fluorescence fluctuations (Zhao et al., 2022). Further, a number of other image analysis-based super-resolution techniques available as Fiji/ImageJ plug-ins, such as multiple signal classification algorithm (MUSICAL) and mean-shift super-resolution (MSSR) also have been reported in rapid succession (Alva et al., 2022; Torres-García et al., 2022). These novel image analysis-based super-resolution techniques may also be possible for application to *in vivo* super-resolution imaging in the future. However, at present, these techniques cannot be applied directly to two-photon observation, and future adaptations are needed. We hope that the validation and optimization know-how for the application of the image analysis-based high-resolution technique to two-photon microscopy reported in this study will help in this regard.

### 4.2. Image analysis artifacts

Suppressing artifacts occurring during numerical operations is critical for image-analysis-based methodologies, including the SRRF method (Burgert et al., 2015; Demmerle et al., 2017). In the present study, we observed noticeable artifacts after applying SRRF processing to images with pixel sizes not meeting the Nyquist frequency (Supplementary Figure S1). A previous study reported that both spatial and temporal correlation analysis parameters in SRRF processing were critical for the spatial resolution and morphological reproducibility of the reconstructed images (Gustafsson et al., 2016). In the study, for the evaluation of SRRF artifacts, they used ground truth images obtained by SMLM observation. However, it is usually difficult to prepare such ground truth for the actual observation of biological specimens. Recently, several evaluation methods, such as SQUIRREL (Culley et al., 2018) and DETECTOR (Gao et al., 2021), have been used to detect the artifact of reconstructed super-resolution images. These methods use image correlation between non-super-resolution and super-resolution images of the same field of view. Enhanced SRRF (eSRRF), an improved version of SRRF processing, also implements automatic optimization of processing parameters based on SQUIRREL and spatial resolution evaluation using FRC analysis (Laine et al., 2022). If there was no ground truth, these methods would help to ensure that the reconstructed microstructures are not artifacts.

In the present study, we used SIM images as the ground truth. 2P-SRRF observations were performed in the same field of view as SIM to ensure morphological reproducibility (Figures 1, 4). In addition, we optimized the parameters for SRRF processing via comparison using the SQUIRREL method (Supplementary Figure S2). The optimum set of parameters successfully suppressed the artifacts resulting from 2P-SRRF. Together, we could achieve low-artifact high-resolution imaging.

### 4.3. Temporal resolution of 2P-SRRF

The morphology of living organisms at the sub-micrometer level often changes within seconds. However, existing super-resolution microscopy techniques require more acquisition time than conventional fluorescence microscopy. For SMLM, the acquisition time requires minutes if not hours. Meanwhile, SRRF requires only several 10 sequential frames for the reconstruction of a super-resolution image and is therefore considered a high-throughput super-resolution method (Gustafsson et al., 2016). In the present study, the acquisition time for the sequential frames obtained for SRRF processing was several seconds (see Materials and Methods, Supplementary Table S1). Even at this throughput of imaging (6 s per a reconstructed image), 2P-SRRF allows the observation of fine neuron structures *in vivo* (Figure 5) due to spine morphology changes being much slower than the 2P-SRRF throughput (Kashiwagi et al., 2019). Nonetheless, to observe microtubules, actin, mitochondria, and other cytoskeleton and intracellular organelles that change more rapidly, a higher throughput is needed. To this end, we previously developed a fast two-photon imaging system using a Nipkow disk to visualize biological phenomena that occur at high speeds, such as microtubule rearrangements during cell division (Otomo et al., 2015; Sasaki et al., 2019). In combination with such a two-photon high-speed imaging system, 2P-SRRF may achieve even faster deep high-resolution imaging.

### 4.4. Possibility of deeper imaging

2P-SRRF imaging was useful for the observation of fluorescent beads embedded in a biomimetic gel even at a depth of 1,500 μm (Figure 2B). In the present study, the cortical layer 5 neurons with well-developed dendrites were used to demonstrate the visualization of spine morphology on *in vivo* imaging with 2P-SRRF (Figure 5). Based on the result of biomimetic gel experiment, we expect that deeper *in vivo* 2P-SRRF observations are also possible. Of course, there are heterogeneities in the living brain, such as white matter, which are not present in gels. Also, the depth-reachability of *in vivo* imaging varies depending on the biological conditions such as the age of the mouse, the state of hemorrhage during surgery, and the region of the brain to be observed. By optimizing such biological conditions, suppressing optical aberrations, and applying a high-peak power excitation laser, previously, we successfully visualized the hippocampal dentate gyrus (at 1,600 μm depth from the brain surface) *in vivo* mouse brain (Kawakami et al., 2015). In the observation, images were obtained with SNR ≈10 even in the hippocampal region beyond the white matter. The single image acquisition time was also similar to that for the acquisition of gel-embedded beads in the present study, within a few seconds. Taken together, these facts suggest that *in vivo* high-resolution imaging of the hippocampus utilizing 2P-SRRF is potentially possible.

### 4.5. Limitations of research

SRRF processing can only improve spatial resolution in the planar direction (Gustafsson et al., 2016). Therefore, our method is also currently only suitable for two-dimensional observations. An extension of the method for three-dimensions will be needed for further application to neuroscience such as the measurement of dendric spine volume. The three-dimensional processing of SRRF has been partially realized by utilizing multi-focus microscopy (Laine et al., 2022). For the application of 2P-SRRF in three-dimensions, further high-throughput image acquisition or volumetric imaging would be necessary.

## 5. Conclusion

In summary, this study successfully demonstrated the applicability of 2P-SRRF to deep biological high-resolution imaging. Researchers using existing two-photon microscopes may promptly apply 2P-SRRF to expand the range of possible applications for high-resolution observation to deeper areas.

## Data availability statement

The raw data supporting the conclusions of this article will be made available by the authors, without undue reservation.

## Ethics statement

The animal study was approved by Institutional Animal Care and Use Committee of the National Institute of Natural Sciences. The study was conducted in accordance with the local legislation and institutional requirements.

## Author contributions

MT and TN contributed to the conception and design of the study. MT and TT performed experiments, partially supported by KK. MT wrote the first draft of the manuscript. All authors contributed to the article and approved the submitted version.
