# Supplementary material for: Fluorescence radial fluctuation enables two-photon super-resolution microscopy
Source: Front Cell Neurosci. 2023 Oct 10;17:1243633. doi: 10.3389/fncel.2023.1243633 (PMC10595032; doi:10.3389/fncel.2023.1243633)
Supplement: Supplementary file 1 [file Data_Sheet_1.pdf]

Supplementary Material

Fluorescence Radial Fluctuation Enables Two-Photon Super-Resolution Microscopy

Motosuke Tsutsumi, Taiga Takahashi, Kentaro Kobayashi, Tomomi Nemoto*

*** Correspondence:** Tomomi Nemoto: tn@nips.ac.jp

Supplementary Figures and Tables

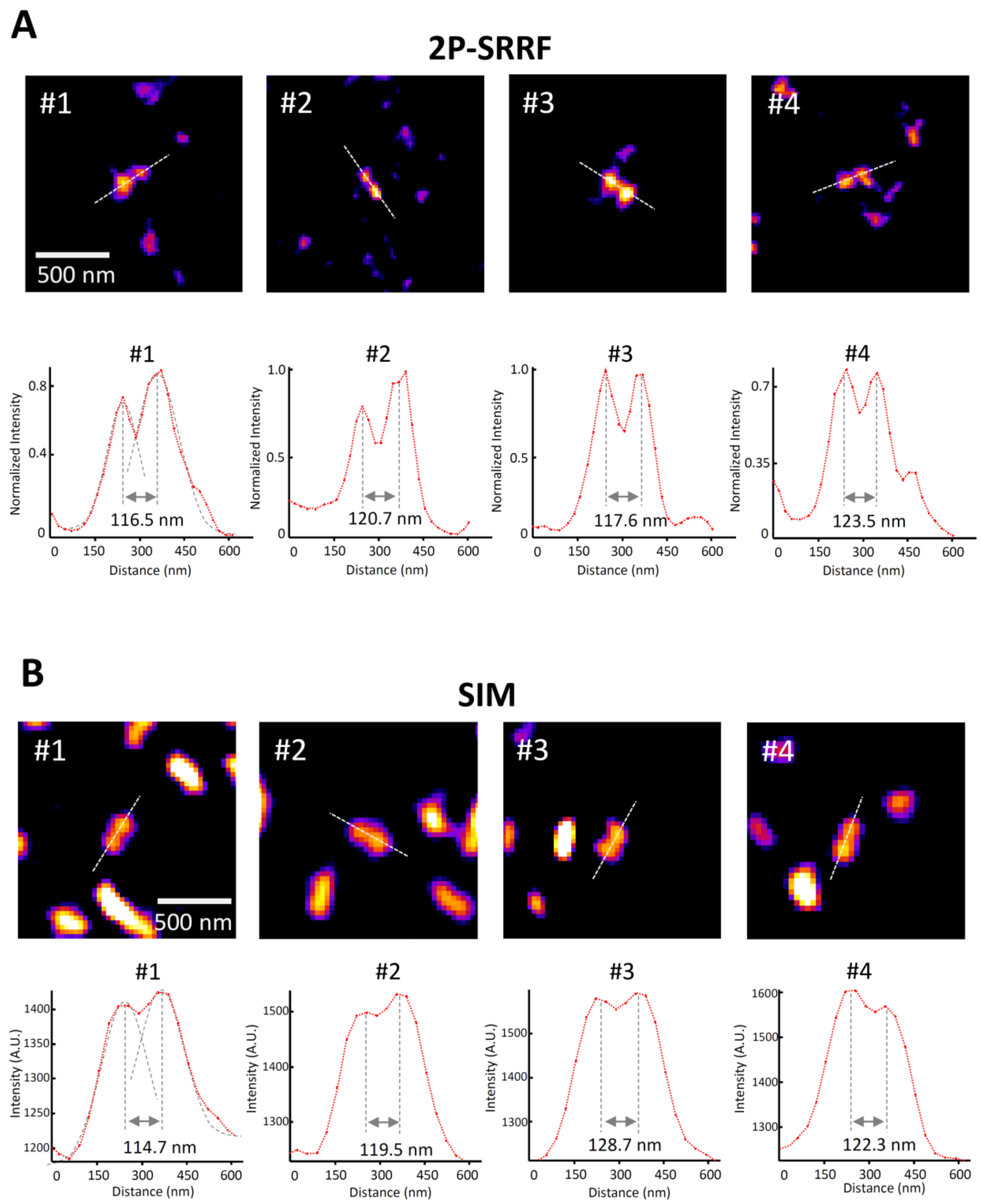

Supplementary Figure 1. Comparisons of 120 nm-nanoruler images between 2P-SRRF and SIM. Different examples of images and their respective intensity profiles of 2P-SRRF **(A)** and SIM **(B)** on the same nanoruler slide sample as in Figure 1. Each profile (red) shows the fluorescence intensity distribution along the white dashed line indicated in the images. The peak-to-peak distance was calculated as the distance between the vertices of two peaks determined by Gaussian curve fitting.

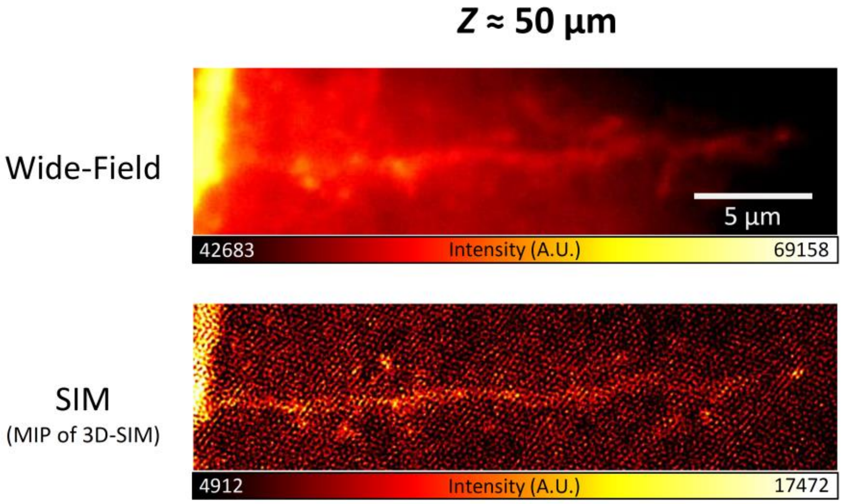

Supplementary Figure 2. Difficulty of SIM observation in opaque brain tissue. Wide-field and SIM observation of the basal dendrite of layer 5 pyramidal cell at a depth of 50 μm in the same brain slice as in Figure 3.

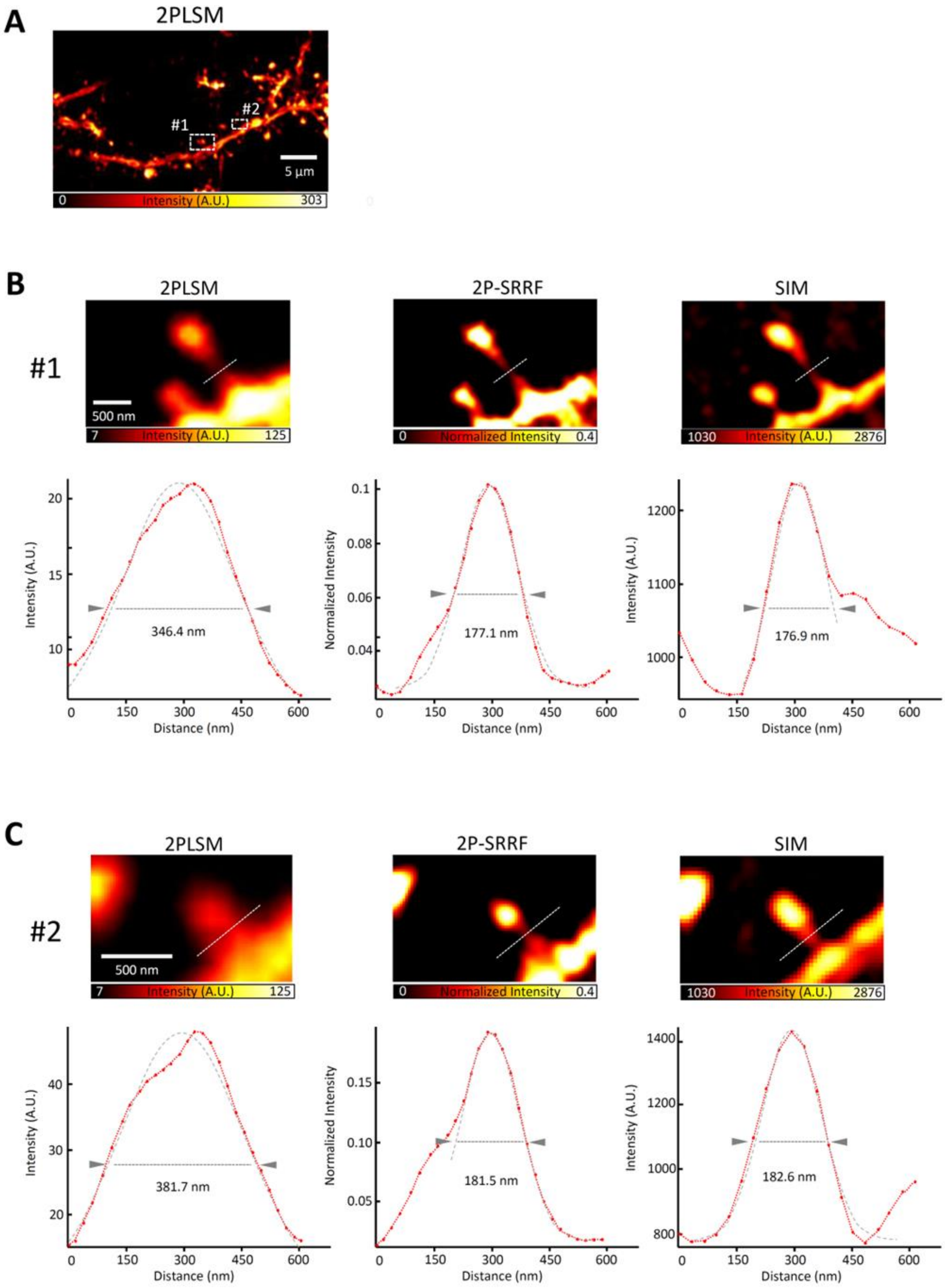
A
2PLSM
#1
#2
5 µm
0 Intensity (A.U.) 303
B
2PLSM
2P-SRRF
SIM
#1
500 nm
7 Intensity (A.U.) 125
0 Normalized Intensity 0.4
1030 Intensity (A.U.) 2876
Intensity (A.U.)
Normalized Intensity
Distance (nm)
346.4 nm
177.1 nm
176.9 nm
C
#2
381.7 nm
181.5 nm
182.6 nm

Supplementary Figure 3. Comparisons of reproducibility of tiny spine structures between 2P-SRRF and SIM. Different examples of dendritic spines observed by 2P-SRRF and SIM on the same dendrite as in Figure 4. Two regions indicated by white dashed lines were selected **(A)**. Enlarged images and their respective intensity profiles of the regions **(B, C)**. Each profile (red) shows the fluorescence intensity distribution along the white dashed line indicated in the images. Arrowheads in the intensity profiles indicate the FWHM calculated by Gaussian curve fitting.

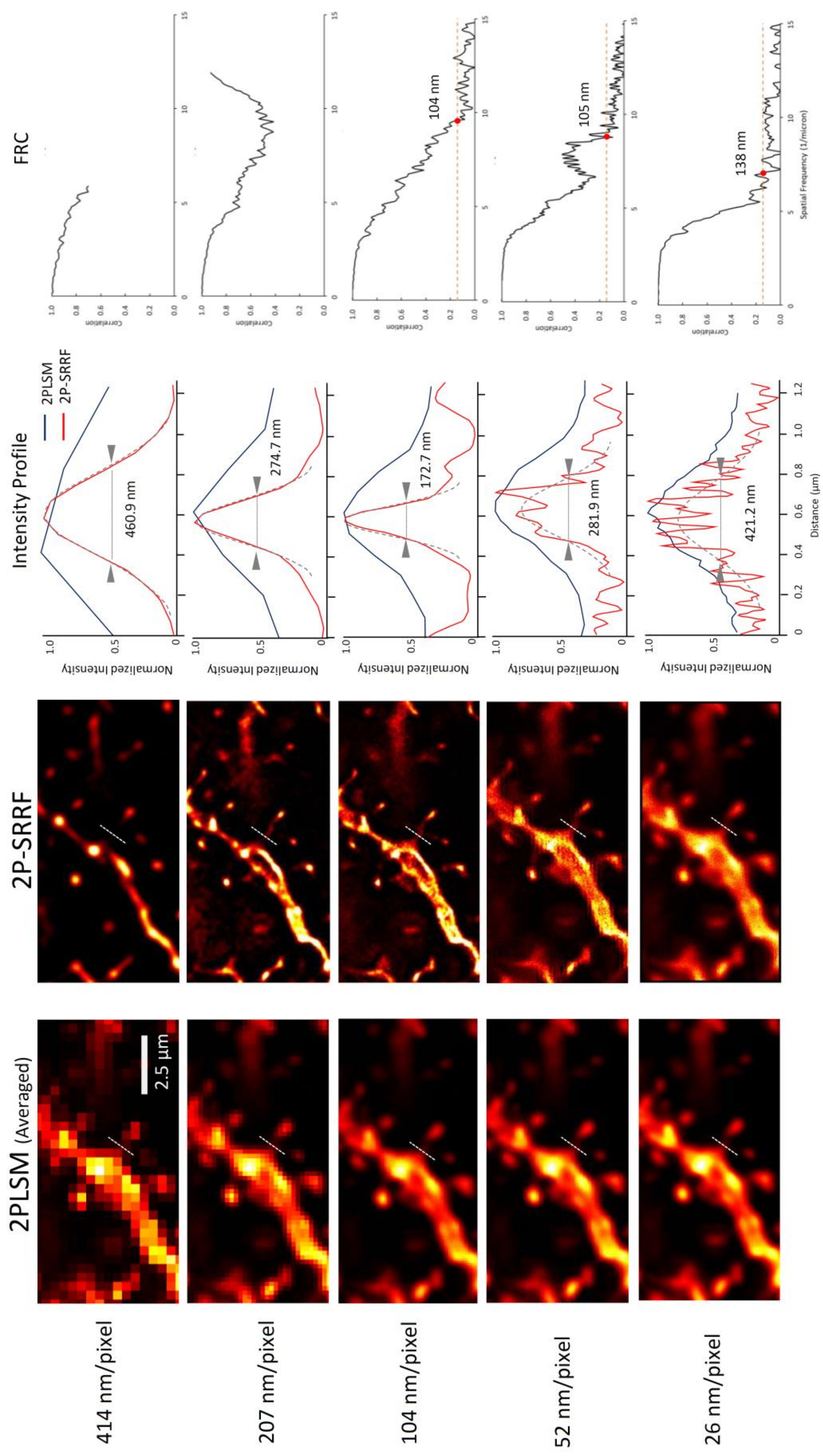
2PLSM (Averaged)
2P-SRRF
Intensity Profile
FRC
2.5 µm
414 nm/pixel
207 nm/pixel
104 nm/pixel
52 nm/pixel
26 nm/pixel
2PLSM
2P-SRRF
460.9 nm
274.7 nm
172.7 nm
281.9 nm
421.2 nm
Normalized Intensity
Distance (µm)
104 nm
105 nm
138 nm
Correlation
Spatial Frequency (1/micron)

Supplementary Figure 4. Effect of pixel size on 2P-SRRF processing. 2P-SRRF and averaged original two-photon images of the basal dendrites of layer 5 pyramidal cells near the brain surface in a fixed coronal slice of Thy1-EYFP H mouse. Two-photon averaged images and 2P-SRRF images acquired at different pixel sizes. Comparison of intensity profiles of identical spine necks along the dashed white lines in the images and Fourier ring correlations of 2P-SRRF images at each pixel size.

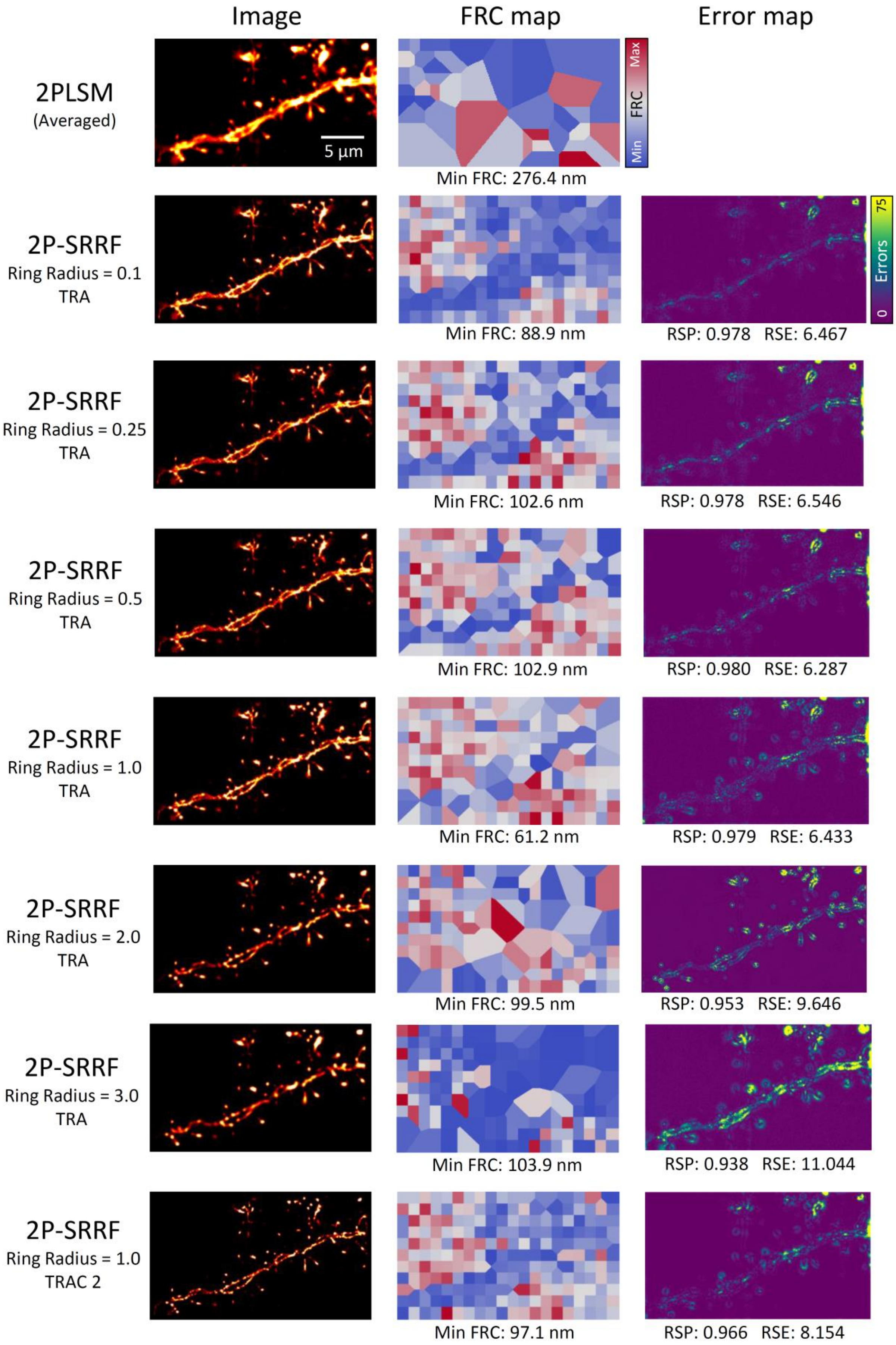
Image
FRC map
Error map
2PLSM
(Averaged)
5 µm
Max
FRC
Min
Min FRC: 276.4 nm
2P-SRRF
Ring Radius = 0.1
TRA
Min FRC: 88.9 nm
75
Errors
0
RSP: 0.978 RSE: 6.467
2P-SRRF
Ring Radius = 0.25
TRA
Min FRC: 102.6 nm
RSP: 0.978 RSE: 6.546
2P-SRRF
Ring Radius = 0.5
TRA
Min FRC: 102.9 nm
RSP: 0.980 RSE: 6.287
2P-SRRF
Ring Radius = 1.0
TRA
Min FRC: 61.2 nm
RSP: 0.979 RSE: 6.433
2P-SRRF
Ring Radius = 2.0
TRA
Min FRC: 99.5 nm
RSP: 0.953 RSE: 9.646
2P-SRRF
Ring Radius = 3.0
TRA
Min FRC: 103.9 nm
RSP: 0.938 RSE: 11.044
2P-SRRF
Ring Radius = 1.0
TRAC 2
Min FRC: 97.1 nm
RSP: 0.966 RSE: 8.154

Supplementary Figure 5. Parameter sweep of various ring radius values on 2P-SRRF processing. Reconstructed images of the basal dendrites of layer 5 pyramidal cells near the brain surface in a fixed coronal slice of Thy1-EYFP H mouse treated by SRRF using various ring radius values. Corresponding FRC maps and reconstruction error maps to each reconstructed image were calculated by using NanoJ-SQUIRREL. TRA means the temporal radiality average, a temporal analysis method for SRRF treatment. TRAC means temporal radiality auto-correlations, which is also a temporal analysis method for SRRF treatment. Min FRC means the minimum value of FRC in each FRC map. RSP means the resolution scaled Pearson coefficient between the reference (averaged 2PLSM image) and 2P-SRRF images. RSE means the resolution scaled error between them.

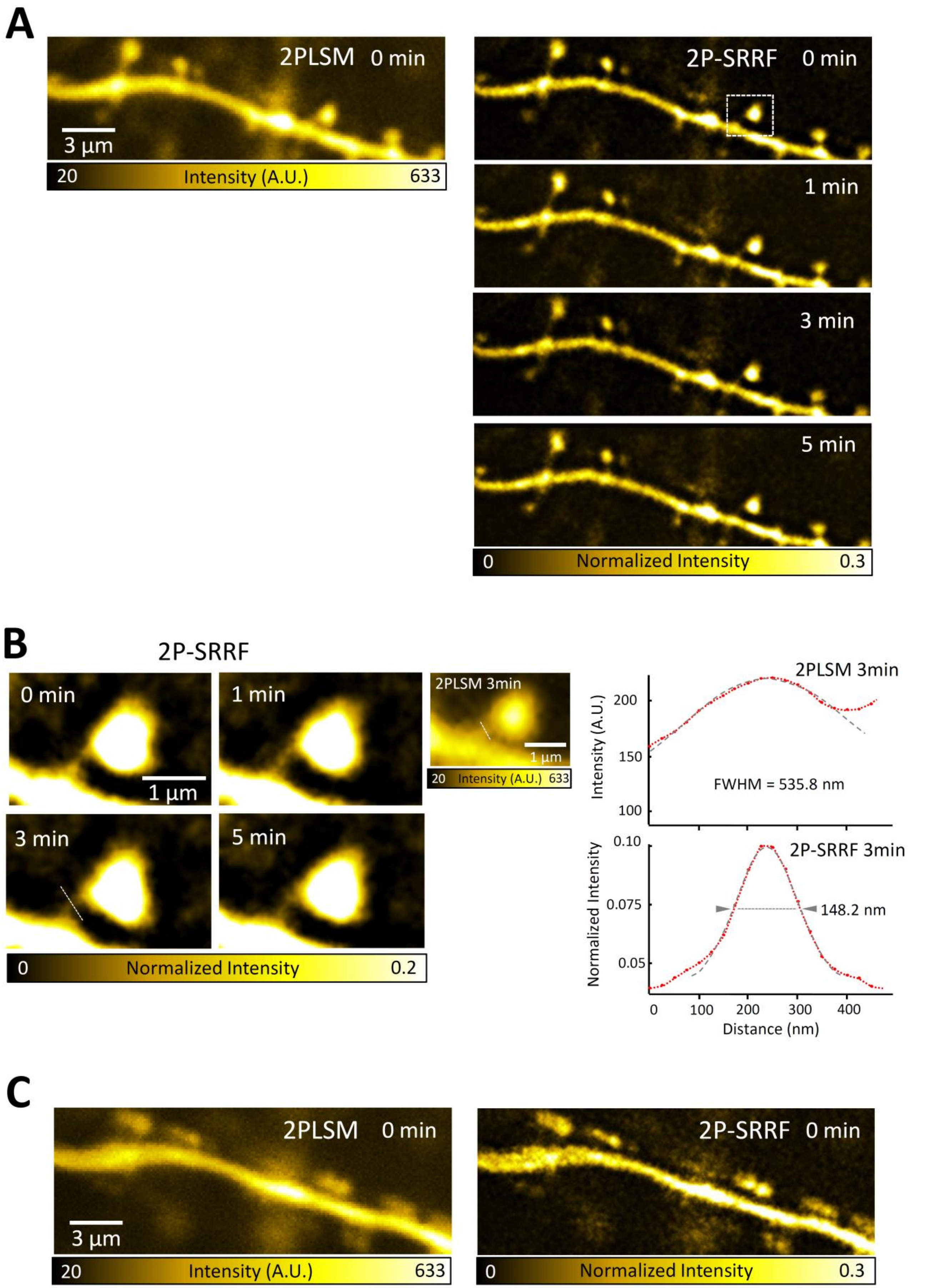
A
2PLSM 0 min
3 µm
20 Intensity (A.U.) 633
2P-SRRF 0 min
1 min
3 min
5 min
0 Normalized Intensity 0.3
B
2P-SRRF
0 min
1 min
3 min
5 min
1 µm
0 Normalized Intensity 0.2
2PLSM 3min
1 µm
20 Intensity (A.U.) 633
2PLSM 3min
Intensity (A.U.)
FWHM = 535.8 nm
2P-SRRF 3min
Normalized Intensity
148.2 nm
Distance (nm)
C
2PLSM 0 min
3 µm
20 Intensity (A.U.) 633
2P-SRRF 0 min
0 Normalized Intensity 0.3

Supplementary Figure 6. Timelapse *in vivo* 2P-SRRF imaging. (A) 2P-SRRF and averaged original two-photon images of the dendrites of layer 2 of the visual cortex at 100 µm depth in a living Thy1-EYFP H mouse brain. For timelapse 2P-SRRF imaging, 30 consecutive images were taken at 0, 1, 3, and 5 minutes after the start of the observation. Before SRRF treatment, consecutive images were corrected the motion blur by using TurboReg treatment. (B) Enlarged image of the region surrounded by a dashed white line in A. The intensity profiles of a spine neck along the dashed white lines in 2P-SRRF and original 2PLSM images were shown on the right side. Arrowheads in the intensity profiles indicate the FWHM calculated by Gaussian curve fitting. (C) 2PLSM and 2P-SRRF images without motion blur registration. In this case, averaging or SRRF treatment was performed without TurboReg treatment.

Supplementary Table 1. Imaging Conditions

Observation	Objective	Laser power	Scan speed	Acquisition time for a single SRRF image
**Nanoruler (Fig. 1)**	60×	4.0%	4 fps	7.5 s
**Brain mimetic gel, @ 500 µm (Fig. 2A)**	25×	25%	27 fps	1.1 s
**Brain mimetic gel, @ 1500 µm (Fig. 2B)**	25×	100%	0.8 fps	37.5 s
**Fixed brain tissue (Fig. 3, 4, S1)**	60×	0.80%	4 fps	7.5 s
***In vivo* mouse brain imaging (Fig. 5)**	25×	22.4%	5 fps	6.0 s
***In vivo* timelapse mouse brain imaging (Fig. S3)**	25×	10%	15 fps	2.0 s
